# Environmental heterogeneity influences liana community differentiation across a Neotropical rainforest landscape

**DOI:** 10.1002/ece3.11170

**Published:** 2024-03-24

**Authors:** Iván Leonardo Ek‐Rodríguez, Jorge A. Meave, Armando Navarrete‐Segueda, M. Lourdes González‐Arqueros, Guillermo Ibarra‐Manríquez

**Affiliations:** ^1^ Laboratorio de Ecología y Sistemática Vegetal, Instituto de Investigaciones en Ecosistemas y Sustentabilidad Universidad Nacional Autónoma de México Morelia Michoacán Mexico; ^2^ Posgrado en Ciencias Biológicas Universidad Nacional Autónoma de México Coyoacán Ciudad de México Mexico; ^3^ Departamento de Ecología y Recursos Naturales, Facultad de Ciencias Universidad Nacional Autónoma de México Coyoacán Ciudad de México Mexico; ^4^ CONAHCYT‐Instituto de Investigaciones en Ciencias de la Tierra Universidad Michoacana de San Nicolás de Hidalgo Morelia Michoacán Mexico

**Keywords:** climbing plants, community assembly, floristic differentiation, habitat associations, structural heterogeneity, topo‐edaphic habitats

## Abstract

We examined the variation in liana community composition and structure across geopedological land units to test the hypothesis that environmental heterogeneity is a driving force in liana community assembly. The study site was the Los Tuxtlas Tropical Biology Station, SE Mexico, a reserve that encompasses 640 ha of tropical rainforest. We sampled all lianas with basal diameter ≥1 cm in three 0.5‐ha plots established in each of five land units (totaling 15 plots and 7.5 ha). We censused 6055 individuals and 110 species. Overall, the most speciose families were also the most abundant ones. Density and basal area of some dominant liana species differed among land units, and a permutational multivariate analysis of variance (PERMANOVA) and a non‐metric multidimensional scaling ordination (NMDS) revealed differences in the presence, density, and basal area of liana species across the landscape. Liana composition and structure were highly heterogeneous among land units, suggesting that variations in soil water availability and relief are key drivers of liana community spatial differentiation. By showing that soil and topography play an important role at the landscape scale, we underscore the ecological relevance of environmental heterogeneity for liana community assembly. In the future, as our ability to assess the local environmental complexity increases, we will gain a better understanding of the liana community assembly process and their heterogeneous distribution in tropical forests.

## Graphical abstract

By analyzing liana community variation among land units (LUs) that differ in geomorphology and soil properties along a 640‐ha tropical rainforest landscape in a neotropical reserve, we found strong evidence supporting our hypothesis that environmental heterogeneity, as represented by discrete LUs, promotes differentiation in liana composition across the landscape. We discuss the factors associated with liana community variation, the potential limitations of the landscape stratification approach to detecting these associations, and the implications for future studies aiming to better understand the assembly of the liana communities.

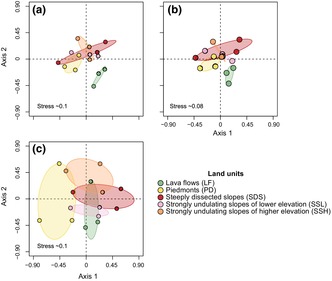

## 1 INTRODUCTION

An overarching goal of vegetation ecology is to explain spatial patterns of plant community composition, structure, and diversity. Most theoretical models aimed to predict species distributions and variation in community attributes are related to the environmental filtering hypothesis (Kraft et al., 2015; Vellend, 2016). This hypothesis rests on the principle that species differ in habitat requirements (i.e., niche differences), and therefore, their establishment and survival in communities are driven by multiple abiotic factors and biotic interactions (Kraft & Ackerly, 2014). Consequently, environmental heterogeneity, that is, the spatial variation in abiotic factors, can promote differentiation in plant performance, which in turn is reflected in forest composition and structure variation (Châve, 2008; Jucker et al., 2018; Stein et al., 2014). Despite the abundant literature examining the role of environmental heterogeneity in the spatial differentiation of tropical forests, it is noteworthy that most studies have focused on their tree component (Châve, 2008; Durán et al., 2006; Fayolle et al., 2012; Guo et al., 2017; Jucker et al., 2018; Navarrete‐Segueda et al., 2021). Due to this bias, these studies only provide a partial understanding of the community assembly process and forest dynamics, considering the different functional plant groups that characterize these forests.

The liana growth form is diverse and represents a conspicuous woody component of tropical forests. In the Neotropics, lianas account for ca. 40% of woody stems although they only contribute with ca. 5% of community basal area (Schnitzer, 2018; Schnitzer & Bongers, 2002). Lianas also account for more than 20% of species richness, and in Neotropical lowland forests, Bignoniaceae and Fabaceae are often the families with the largest species diversity (Gentry, 1991). In addition to these regional patterns, liana community composition and structure develop highly variable and complex habitat associations across heterogeneous landscapes (Addo‐Fordjour et al., 2014; DeWalt et al., 2006; Ibarra‐Manríquez & Martínez‐Ramos, 2002; Macía, 2011). However, the multifactorial nature of the relationship between lianas and their environment challenges our ability to explain patterns in liana community attributes.

Broad‐scale studies have shown that annual precipitation and dry season length are key regional drivers of liana biomass, density, and diversity (DeWalt et al., 2010, 2015; Parolari et al., 2020). Nonetheless, at smaller scales, climatic effects on liana community attributes may be overridden by other factors operating locally, such as topography, soil properties, parent material, tree community structure, and forest dynamics (Dalling et al., 2012; DeWalt et al., 2006; Ibarra‐Manríquez & Martínez‐Ramos, 2002; Ledo & Schnitzer, 2014; Liu et al., 2021, 2023; Pérez‐Salicrup & De Meijere, 2005; Rocha et al., 2022; Schnitzer et al., 2012; Vleut & Pérez‐Salicrup, 2005). This is so because lianas germinate in the soil and always maintain a connection with it while they are mechanically dependent on trees (Gentry, 1991; Ibarra‐Manríquez et al., 2015). Additionally, the effects of local factors could be both direct and indirect because of their complex interactions (Jucker et al., 2018; Liu et al., 2021, 2023), highlighting the need to disentangle their effects through an appropriate approach.

Geomorphological variation is expected to promote differentiation in liana communities through the creation of mosaics of potential habitats (Florinsky, 2016; Hulshof & Spasojevic, 2020; Turner & Gardner, 2015; Zoneveld, 1989). Geomorphological factors (e.g., slope aspect and inclination, elevation, parental material) drive variation in soil properties and thus may influence plant community assembly (Baldeck et al., 2013; Durán et al., 2006; Guo et al., 2017; Rodrigues et al., 2019). Numerous studies in tropical forests have shown evidence that tree communities respond to the environmental heterogeneity associated with geomorphology and soils (e.g., Clark & Clark, 2000; Denslow et al., 2019; Durán et al., 2006; Fayolle et al., 2012; Guo et al., 2017; Jucker et al., 2018; Muñoz et al., 2023; Navarrete‐Segueda et al., 2021). However, studies focusing on liana communities are scarce (Addo‐Fordjour et al., 2014; DeWalt et al., 2006; Ibarra‐Manríquez & Martínez‐Ramos, 2002). Therefore, the examination of the relationship between tropical liana communities and the environmental heterogeneity related to geomorphology enables a multivariate integration of the factors influencing their assembly and their comprehensive understanding and thus provides valuable information about species distributions, habitat associations, and community structure.

Here, we asked if liana community attributes vary according to a landscape stratification based on landforms, geology, and soil features. The territory of the Los Tuxtlas Tropical Biology Station (LTBS) was used as a study system where the climate is relatively homogeneous, but geomorphology is highly variable. Navarrete‐Segueda et al. (2021) distinguished several landscape units (LUs) that characterize the environmental mosaic found in this protected area; this mosaic may underlie patterns of liana community differentiation, a growth form scarcely known in this reserve. We hypothesized that the environmental mosaic promotes spatial differentiation at the community level, in terms of species composition and community structure, by representing contrasting habitat combinations for liana species. The aims of this study were twofold: (1) to assess the composition and structure of the liana communities occurring across the LTBS, and (2) to analyze the influence of landscape‐scale environmental heterogeneity on these attributes.

## 2 METHODS

### 2.1 Study area

**FIGURE 1:**
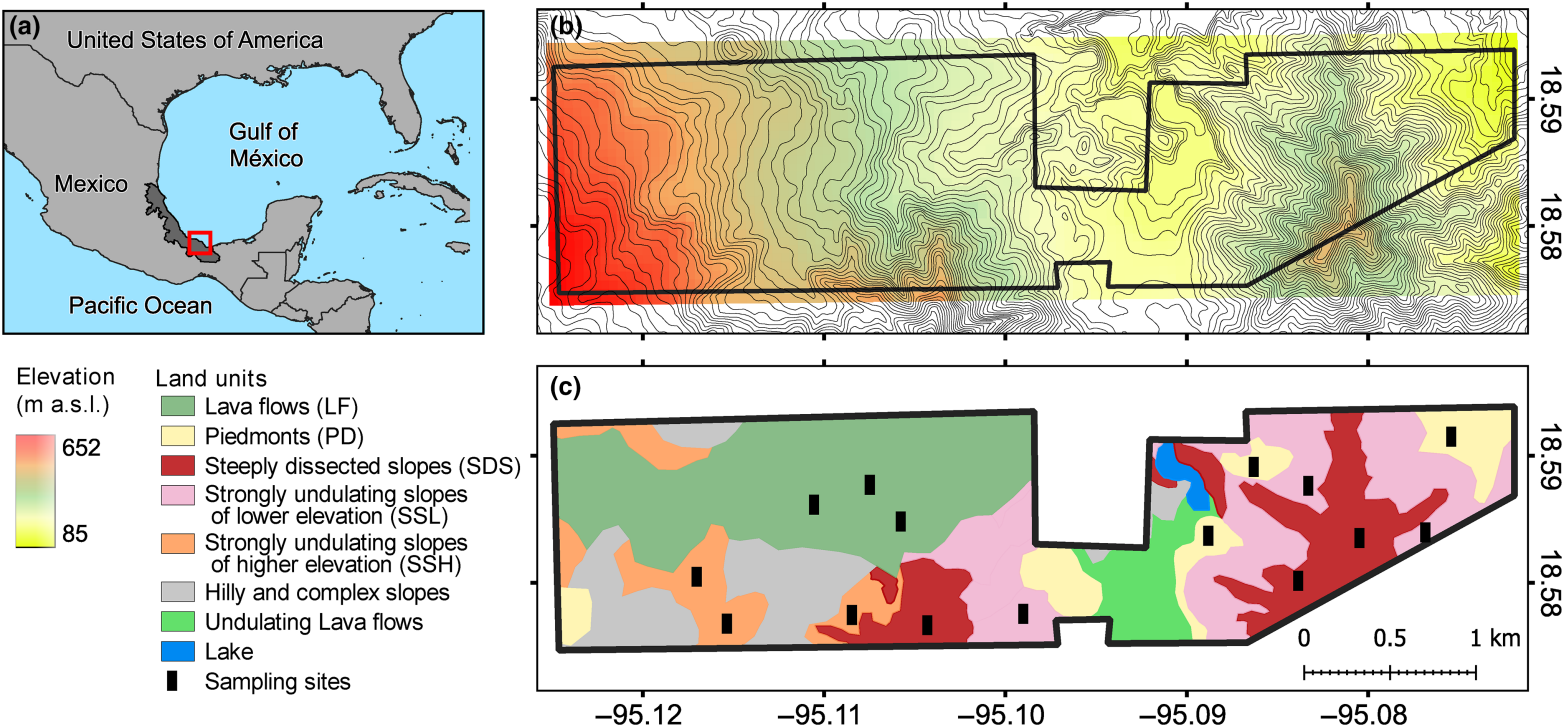
Maps of the Los Tuxtlas Tropical Biology Station, Mexico. (a) Location of the study area in Veracruz State, Mexico (red square). (b) Contour map showing elevation range. (c) Landscape stratification of land units (see Navarrete‐Segueda et al., 2021 for details on land unit delimitation) showing the location of the plots used for surveying the liana community.

The LTBS is located on the eastern slope of the San Martín Tuxtla volcano (Los Tuxtlas Range), Veracruz State, Mexico (Figure 1). The region has a long history of use and management, which is reflected in its high fragmentation due to extensive human activities. Nonetheless, the LTBS shelters 640 ha of old‐growth tropical rainforest and is one of the most studied reserves in the country. The climate is tropical and humid, with abundant rainfall in most of the year and a short relatively dry season in April and May. According to data from the meteorological station of the LTBS covering the 2011–2017 period, the mean annual temperature is 24.2°C, with a maximum monthly normal of 30.4°C (May) and a minimum of 18.1°C (January); total annual precipitation is 3433 mm on average, with a mean monthly maximum of 551 mm in October and a mean monthly minimum of 78 mm in April (Ek‐Rodríguez et al., 2022).

Geomorphology and soils are heterogeneous across the LTBS as it is located in a rugged volcanic region (García‐Aguirre et al., 2010). Soils have developed mainly over plagioclase‐rich lava flows (Verma et al., 1993) interbedded with plagioclase, augite, and olivine‐rich tephra from Pleistocene and Holocene cinder cones (Nelson & González‐Caver, 1992). Our study encompasses a range of soil textures (clay to sandy clay loam textures), coarse fragment contents, and depths, indicating differences in soil water and nutrient availability (Miranda‐Gallegos et al., 2023; Navarrete‐Segueda et al., 2021; Table 1). In addition, these conditions interact with an elevational range (ca. 100–650 m a.s.l.), which may influence precipitation across the landscape (Gutiérrez‐García & Ricker, 2011). Slope steepness is also highly variable, ranging from 6° to 34° (Figure 1).

**TABLE 1:** Topo‐edaphic features across five geopedological land units in the tropical rainforest of the Los Tuxtlas Tropical Biology Station, Mexico.

	Lava flows	Piedmonts	Steeply dissected slopes	Strongly undulating slopes	
				Low elevation	High elevation
Slope steepness (°)	Flat (<10°)	Flat (<10°)	Very steep (>25°)	Steep (15–25°)	Steep (10–16)
Elevation (m a.s.l.)	295–403	140–260	393–404	219–320	437–511
Top‐soil organic C (%) (mean ± SD)	15.5 ± 6.0	5.4 ± 0.9	7.6 ± 1.8	6.5 ± 2.4	12.5 ± 1.8
AWHC (L m^−2^) (mean ± SD)	4.0 ± 0.6	88.7 ± 14.7	123.9 ± 30.2	94.14 ± 20.7	77.7 ± 13.5
Parental material	Basanite and alkali basalt	Volcanic ash	Volcanic ash	Volcanic ash	Volcanic ash
Soil type (Reference group)	D: Leptosols; A: Histosols	D: Phaeozems; C: Luvisols	D: Andosol	C: Phaeozems; C: Luvisols; A: Andosol	D: Phaeozems; C: Andosol

*Note*: Slope, elevation, top‐soil (A Horizon) organic C and available water holding capacity (AWHC) were modified from Navarrete‐Segueda et al. (2021) and Miranda‐Gallegos et al. (2023) accordingly to the three 0.5‐ha plots established in each land unit. Parent material is reported according to Nelson and González‐Caver (1992). Soil type was determined for each land unit based on field data and following the IUSS Working Group (2015) classification system. When a soil type covered ≥50, ≥ 25, or ≥5% of the soil surface, it was classified as Dominant (D), Codominant (C), or Associated (A) soil type, respectively.

### 2.2 Geopedological landscape stratification

The spatial variation of soil types and other habitat features allowed Navarrete‐Segueda et al. (2021) to differentiate LUs across the landscape at the LTBS. Among them, five LUs stand out because of their high spatial coverage, as together they account for 75% of the study area; these main LUs are lava flows, piedmonts, steeply dissected slopes, and strongly undulating slopes either at lower or higher elevations (Figure 1; Table 1). Lava flows (LF) constitute relatively flat but irregular terrains with exposed basaltic rock and are thus dominated by shallow stony soils (Leptosols) with low water storage capacity and rooting depth. Piedmonts (PD) are relatively flat areas with moderate to high rooting depth soils of fine texture (Phaeozems). In turn, steeply dissected slopes (SDS) are zones with highly inclined relief (up to 60°), with moderate to high rooting depth (Andosols). These three LUs are connected by strongly undulating slopes occurring at either lower (<350 m; SSL) or higher (>400 m; SSH) elevations (Table 1).

### 2.3 Liana census

We used the landscape stratification proposed by Navarrete‐Segueda et al. (2021) to capture the environmental heterogeneity mosaic based on relief, soil, and geology. This mosaic is composed of discrete LUs that may underlie liana community heterogeneity. We conducted liana censuses in three 0.5 ha (20 × 250 m) plots established in each of the five LUs described above (total sampling area of 7.5 ha), with between‐plot distance ranging from ca. 400 to 4550 m (Figure 1). In each plot, we recorded and tagged all liana individuals with basal diameter ≥1 cm; lianas were determined to species in the field by G. I‐M. and the parataxonomist Mr. S. Sinaca‐Colín, both of whom have a four‐decade long experience in floristic studies in the region (Cornejo‐Tenorio et al., 2019; Ibarra‐Manríquez & Sinaca‐Colín, 1995, 1996a, 1996b). We also included those individuals of the following hemi‐epiphytic liana species, whose roots maintain a lifetime connection with the soil: *Marcgravia mexicana*, *Ruyschia enervia*, *Souroubea loczyi* (Marcgraviaceae); *Schlegelia nicaraguensis* (Schlegeliaceae), *Juanulloa mexicana*, and *Solandra maxima* (Solanaceae) (Table A1 in Appendix 1). The main base of every liana individual was located on the soil to verify that it was rooted within the plot and then we measured its basal diameter at the rooting point with a vernier or measuring tape.

### 2.4 Statistical analysis

All analyses were performed in R (R Core Development Team, 2022). To determine the liana community structure, we estimated the frequency (the number of plots in which a given species was recorded), density (the number of liana individuals per hectare), basal area (the sum of the area of stem bases per hectare), and the mean and maximum diameter for each species and per plot. To identify the dominant species, we quantified the density and total basal area of each species. We then arbitrarily selected the 15 species having the highest values of these attributes and compared their relative contribution among LUs.

To compare community structural attributes among LUs, we fitted general linear models (GLMs), including the LU as a fixed factor. We used the Gaussian family for each model and only the absolute density of individuals was transformed to relative density to avoid overdispersion. For each GLM, we verified normality assumptions by applying a Shapiro–Wilk test on the residuals. Since the differential spatial availability of the examined LUs across the study area is another factor of environmental heterogeneity, we also compared field‐weighted density and basal area values among LUs using GLMs. We estimated the total surface of each LU using the software *QGIS* (QGIS Development Team, 2022) and multiplied the fraction of the total area of the reserve that they represent by their respective values of density and basal area. Finally, we applied the *glht* function of the *multcomp* package ver. 1.4‐25 (Hothorn et al., 2008) for a post hoc test among groups in the models that showed significant differences.

To assess if liana distribution mirrors the landscape stratification, we performed a permutational multivariate analysis of variance (PERMANOVA) by setting the LU as a factor and the community distance matrix as the response variable in the function *adonis2*. We obtained *p*‐values for each PERMANOVA by running 999 permutations. A non‐metric multidimensional scaling (NMDS) ordination was performed with the function *metaMDS* to visualize the PERMANOVA results. We used the function *avgdist* to compute average dissimilarity matrices based on the Jaccard index for binary (presence/absence) data and the Bray–Curtis index for continuous (density and basal area) data. These functions are included in the *vegan* package ver. 2.6‐4 (Oksanen et al., 2022).

## 3 RESULTS

### 3.1 Liana community attributes

**FIGURE A1:**
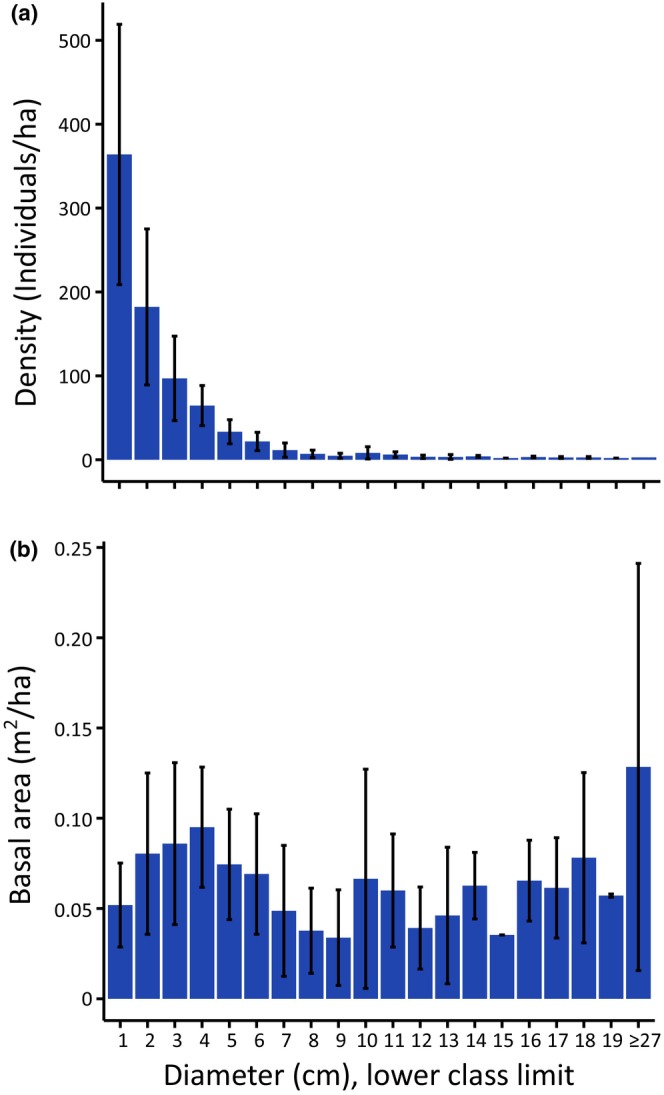
Distribution of (a) density and (b) basal area across diameter classes in the liana community of the rainforest at the Los Tuxtlas Tropical Biology Station, Mexico.

We recorded 6055 liana individuals in the 15 plots (mean ± SD 807.3 ± 267.5 ind. ha^−1^), which comprised a total basal area of 6.61 m^2^ (0.88 ± 0.27 m^2^ ha^−1^); these individuals had a mean diameter of 2.81 cm (2.87 ± 0.45 cm on a per plot basis) and a maximum diameter of 28 cm (18.35 ± 4.66 cm on a per plot basis). The frequency distribution of diameter classes had the reverse J‐shaped curve commonly observed in woody communities; almost half of all liana individuals recorded (47%) had diameters between 1 and 1.9 cm, and their frequency decreased gradually as diameter size increased (Figure A1a in Appendix 1). Basal area was strongly concentrated in only seven individuals with basal diameters ≥27 cm, but lianas with diameters ranging from 2 to 4.9 cm also made an important contribution to community basal area (Figure A1b in Appendix 1).

Among the recorded liana individuals, 110 species, 79 genera, and 35 families were represented (Table A1 in Appendix 1). Almost half (45.5%) of the species belonged to only six families (Table A2 in Appendix 1): Bignoniaceae (17 species), Apocynaceae (7), Malpighiaceae (7), Sapindaceae (7), Celastraceae (6), and Passifloraceae (6); in contrast, 14 families (40%) were represented by a single species. Except for Passifloraceae, the species‐rich families were also important in terms of density (Table A2 in Appendix 1), and in general, they also encompassed the largest numbers of genera: Bignoniaceae (10), Malpighiaceae (6), Apocynaceae, and Celastraceae (5 each). The genera *Passiflora* (6 species), *Fridericia*, *Paullinia*, and *Smilax* (4 species each) had the largest number of species but encompassed only 16.4% of the total density (Table A3 in Appendix 1). These results were consistent among LUs (Tables A2 and A3 in Appendix 1).

### 3.2 Density, basal area, and frequency of species

**FIGURE 2:**
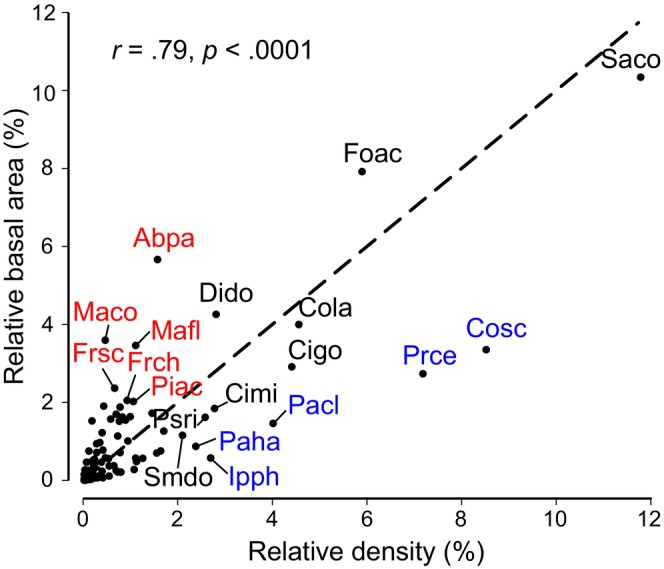
Correlation between relative density and basal area of lianas in the tropical rainforest of the Los Tuxtlas Tropical Biology Station, Mexico. The values for each species were estimated by pooling its density and basal area in fifteen 0.5‐ha plots. The dashed line indicates the identity function (*y* = *x*). Blue dots indicate species whose relative density is at least twice as large as its relative basal area; red dots indicate species whose basal area is at least twice as large as its relative density; black dots indicate species whose ratio between these two structural variables is close to 1. Species acronyms: Abpa, *Abuta panamensis*; Cigo, *Cissus gossypiifolia*; Cimi, *Cissus microcarpa*; Cola, *Combretum laxum*; Cosc, *Connarus schultesii*; Dido, *Dichapetalum donnell*‐*smithii*; Foac, *Forsteronia acouci*; Frch, *Fridericia chica*; Frsc, *Fridericia schumanniana*: Ipph, *Ipomoea philomega*; Maco, *Machaerium cobanense*; Mafl, *Machaerium floribundum*; Pacl, *Paullinia clavigera*; Paha, *Passiflora hahnii*; Piac, *Pisonia aculeata*; Prce, *Pristimera celastroides*; Psri, *Psycopteris rivularis*; Saco, *Salacia cordata*; Smdo, *Smilax domingensis*.

The species with the largest number of individuals were *Salacia cordata* (714 individuals), *Connarus schultesii* (516), and *Pristimera celastroides* (435). In turn, the three species with the highest total basal area were *Salacia cordata* (0.68 m^2^), *Forsteronia acouci* (0.52 m^2^), and *Abuta panamensis* (0.37 m^2^). Together, these species accounted for 27.5% and 23.2% of these variables, respectively. A large proportion of all species (87%) were represented by <100 individuals, and 14 (12.7%) and 7 (6.4%) were singletons or doubletons, respectively. Species density and basal area values were strongly correlated (Pearson product–moment correlation, *r* = .79, df, 108, *p* < .001; Figure 2). Notably, *Salacia cordata*, the species that stands out regarding both attributes, was recorded in all 15 census plots (Table A1 in Appendix 1). Against this correlation, some species had disproportionally higher contributions to basal area because they often have thick stems (e.g., *Abuta panamensis*, *Dichapetalum donnell*‐*smithii*, or *Machaerium cobanense*), while other species were very abundant but often had thin stems, thus contributing mainly to density (e.g., *Ipomoea philomega*, *Passiflora hahnii*, or *Paullinia clavigera*) but little to total basal area. The three individuals having the largest diameters belong to *Dichapetalum donnell‐smithii* (28 cm), *Abuta panamensis* (27 cm), and *Fridericia schumanniana* (26 cm).

**FIGURE A2:**
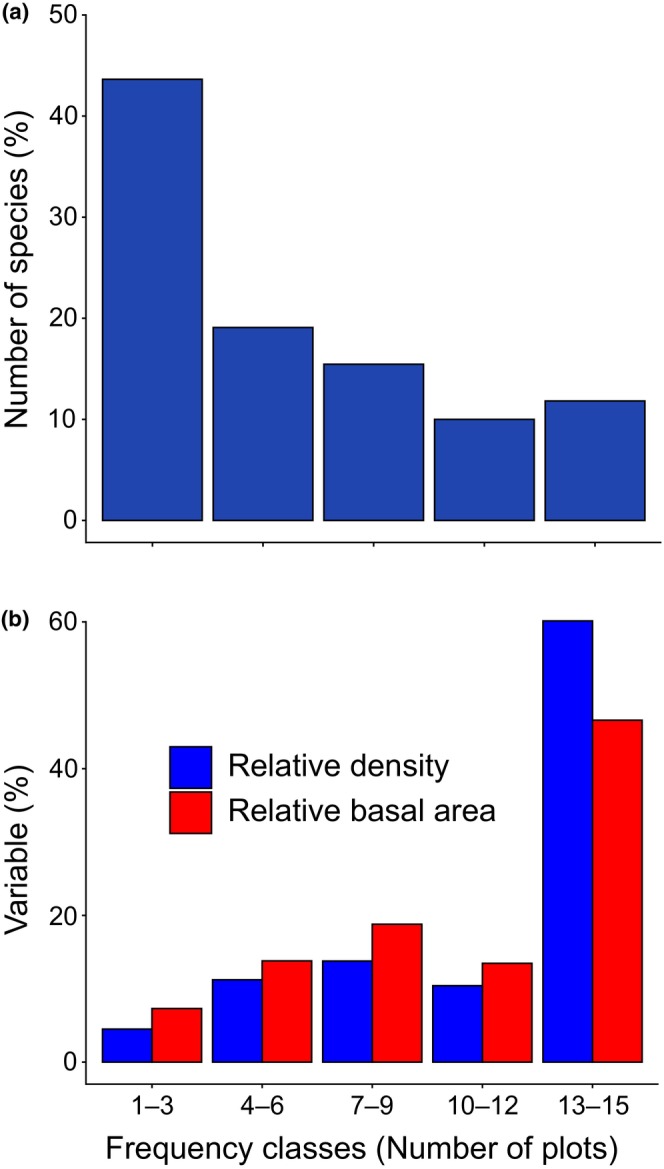
Structural distribution [(a) Species number; (b) Percent of relative density and relative basal area] on frequency classes of lianas in the tropical rainforest of the Los Tuxtlas Tropical Biology Station, Mexico.

Thirteen species (11.8%) were recorded in between 13 and 15 plots (i.e., very frequent species) while 48 species (43.6%) were recorded in three plots or less (i.e., very infrequent species) (Figure A2a in Appendix 1). Together, the very frequent species accounted for 60.1% of total density and 46.6% of total basal area, with a high correlation between these variables and their frequency (*r* = .7 for both, df, 108, *p* < .001). In contrast, the very infrequent species accounted for ca. 0.1% of both variables, together accounting for 4.6% of total density and 7.3% of total basal area (Figure A2b in Appendix 1). Only six species were recorded across all plots (together encompassing 33.3% of all individuals and 29.4% of total basal area); four of them (*Connarus schultesii*, *Forsteronia acouci*, *Paullinia clavigera*, and *Salacia cordata*) were among the top seven in terms of total density (Figure 4a), and three (*Abuta panamensis*, and again *F. acouci*, and *S. cordata*) had the highest basal area values (Figure 4b). The sixth species (*Randia retroflexa*) was not recorded among those lianas having higher density and basal area values (Table A1 in Appendix 1).

### 3.3 Variation of liana community attributes across the landscape

**FIGURE 3:**
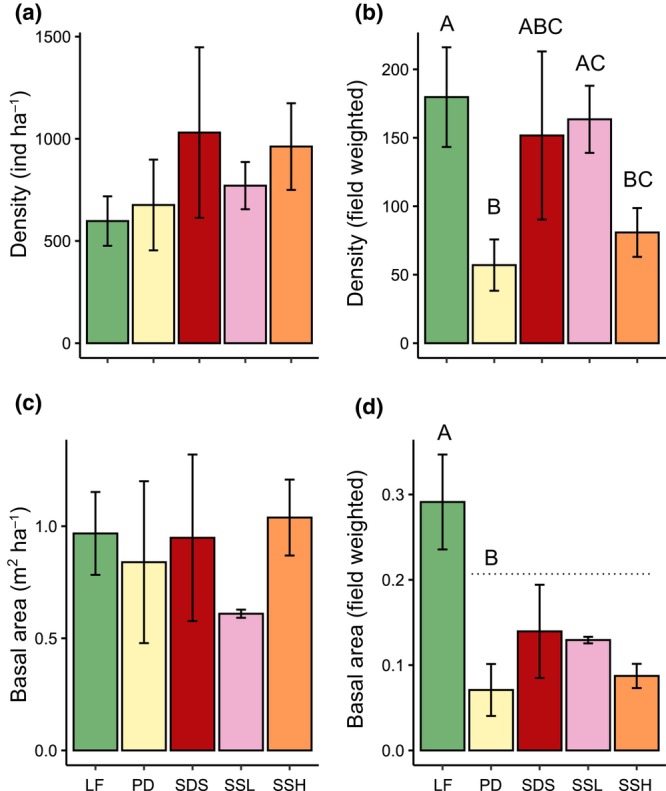
Comparison of density and basal area per hectare (a, c) and their respective field weighted values (b, d) among different land units at the Los Tuxtlas Tropical Biology Station. Bars and arrows indicate means and standard deviations by land unit. Land units: LF, Lava flows; PD, Piedmonts; SDS, Steeply dissected slopes; SSH, Strongly undulating slopes of higher elevation; SSL, Strongly undulating slopes of lower elevation. Capital letters indicate statistical differences between land units based on a post hoc test.

The lava flows were the LU where the lowest liana density was recorded, in strong contrast with SDS, the LU with the highest density, almost twice as large as that of LF (896 vs. 1546 individuals 1.5 ha^−1^, respectively). In contrast to density, liana basal area was less variable among LUs (range: 0.91–1.56 m^2^ 1.5 ha^−1^). Regarding the differences in diameter among LUs, individual mean diameter (2.5–3.3 cm; Table 2) was close to the overall mean (2.81 cm); the largest diameter was recorded in SSH, although LF had the highest number of individuals with diameters ≥10 cm. Against expectations, the fitted GLMs did not show any differences in structural attributes (Table A4 in Appendix 1) among LUs. However, when field weighted density and basal area were compared, strong differences among LUs were revealed (Figure 3), with the highest values corresponding to LF and the lowest ones to PD.

**TABLE 2:** Liana community attributes across five land units in the tropical rainforest of the Los Tuxtlas Tropical Biology Station, Mexico.

Structural attributes	Lava flows	Piedmonts	Steeply dissected slopes	Strongly undulating slopes	
				Low elevation	High elevation
Density (no. ind. in 1.5 ha)	**896**	**1014**	**1546**	**1156**	**1443**
Mean (ha^−1^)	597.3	676.0	1030.7	770.7	962.0
SD	120.9	222.0	417.1	115.4	212.0
Basal area (m^2^ in 1.5 ha)	**1.45**	**1.26**	**1.42**	**0.91**	**1.56**
Mean (ha^−1^)	0.97	0.84	0.95	0.61	1.04
SD	0.18	0.36	0.37	0.02	0.17
Individual mean diameter (cm)	**3.3**	**2.9**	**2.8**	**2.5**	**2.7**
Mean	3.3	2.9	2.9	2.5	2.7
SD	0.2	0.4	0.8	0.2	0.36
Individual maximum diameter (cm)	**21.0**	**26.0**	**16.0**	**17.0**	**28.0**
Mean	20.4	20.3	14.3	14.3	22.3
SD	1.0	5.1	2.1	3.1	5.1
Number of species	**70.0**	**64.0**	**75.0**	**70.0**	**58.0**
Mean (0.5 ha^−1^)	44.0	43.7	42.7	39.0	37.7
SD	5.6	1.5	7.6	2.7	3.2

*Note*: Values are totals (bold typeface) along with means and standard deviations (SD) for the three plots of each land unit. Note that density and basal area values were rescaled to 1 ha, unlike number of species, whose mean an SD are reported per 0.5 ha plot.

**FIGURE 4:**
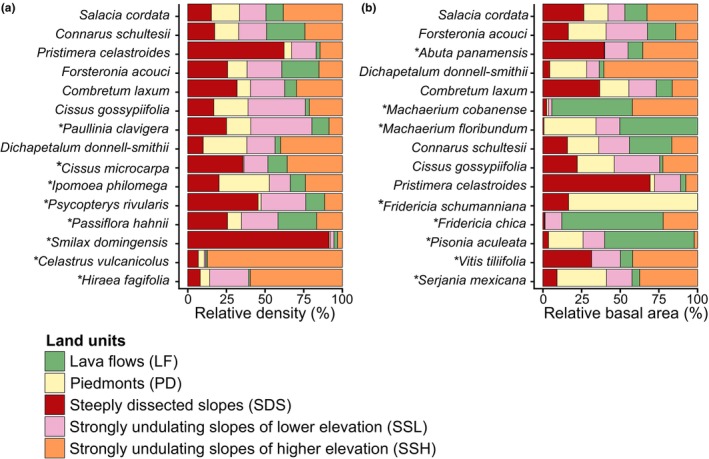
Dominant liana species at the Los Tuxtlas Tropical Biology Station, Mexico. Relative contributions to (a) density and (b) basal area of land units are shown for each species. Species are sorted in decreasing order for both variables. Asterisks indicate those species that are unique to each panel, that is, the dominants for only one variable.

Density and basal area also differed among LUs when the comparison focused on dominant species. The 15 most abundant species (incidentally, all of which had ≥99 recorded individuals), accounted for 65% of total density but some of them had higher densities in some LUs. For example, *Celastrus vulcanicolus* and *Hiraea fagifolia* had their highest densities in SSH, while densities of *Smilax domingensis* and *Pristimera celastroides* were highest in SDS (Figure 4a). In contrast, densities of other species were fairly homogeneous across LUs (e.g., *Salacia cordata*, *Connarus schultesii*, *Forsteronia acouci*). Likewise, the relative contributions of the 15 species with the highest basal area (together accounting for 58.4% of this community attribute) differed among LUs (Figure 4b). This variable was similar across all LUs for the two top species (*Salacia cordata* and *Forsteronia acouci*), but other species had higher basal area values in two LUs (*Fridericia schumanniana* in SDS and PD; *Machaerium cobanense* in SSH and LF), or in one LU only (*Pisonia aculeata* and *Fridericia chica* in LF).

**FIGURE A3:**
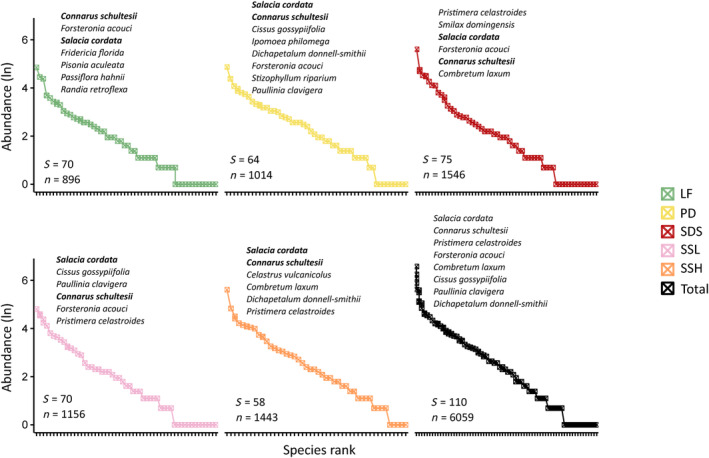
Rank‐order curves showing the relative abundance of species recorded in each land unit and combining the data from the five land units (Total) in the tropical rainforest of the Los Tuxtlas Tropical Biology Station, Veracruz, Mexico. Land units: LF, Lava flows; PD, Piedmonts; SDS, Steeply dissected slopes; SSH, Strongly undulating slopes of higher elevation; SSL, Strongly undulating slopes of lower elevation.

Rare species, that is, those with four individuals or less (30% of all species) were recorded in only one (23 species) or two (10 species) LUs. The LU with more singletons was SSL (17 species), while in SSH only six singletons were recorded (Figure A3 in Appendix 1). Interestingly, the singletons recorded in some LUs were not necessarily rare species across the landscape, since some were among the most abundant species elsewhere (e.g., *Celastrus vulcanicolus*, *Smilax domingensis*, and *Cissus macrocarpa*).

**FIGURE 5:**
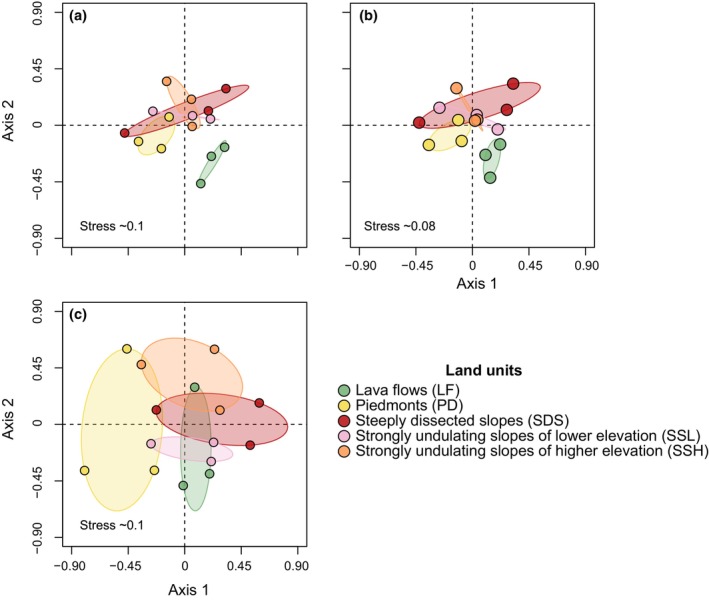
Non‐metric multidimensional scaling ordinations of the liana community at the landscapes of the Los Tuxtlas Tropical Biology Station, Mexico. The ordinations are based on species by plot matrices of (a) presence‐absence, (b) density, and (c) basal area data. Survey plots are showed as color dots indicating the land unit where they are located.

The number of species per LU ranged from 58 in SSH to 75 in SDS (Table 2). The PERMANOVA revealed significant differences for species presence (*F*_4,14_ = 1.13, *R* ^2^ = .31, *p* < .05), density (*F*_4,14_ = 1.2, *R* ^2^ = .32, *p* < .05), and basal area (*F*_4,14_ = 1.4, *R* ^2^ = .36, *p* < .05) among LUs. In turn, the NMDS ordinations showed a different degree of differentiation among LUs (Figure 5). Regarding species presence and density, LF and PD plots were the most strongly segregated from plots in other LUs (Figure 5a,b), while in the basal area‐based ordination, only PD was clearly separated from the other LUs (Figure 5c).

## 4 DISCUSSION

We analyzed liana community variation among LUs that differ in geomorphology and soil properties in the protected landscape of the LTBS. We found strong evidence supporting our hypothesis that environmental heterogeneity, as represented by discrete LUs, promotes differentiation in liana community composition and structure across the landscape. In the following sections, we first compare our results with previous work both at the LTBS and other Neotropical sites, emphasizing the relevance of our approach. We then discuss in detail the factors associated with liana community variation, the potential limitations of the landscape stratification approach to detect these associations, and the implications for future studies aiming to better understand the assembly of liana communities.

### 4.1 Liana community in the LTBS

Our liana sampling approach resulted in the recording of practically all previously known liana species in the study area (Ibarra‐Manríquez & Sinaca‐Colín, 1995, 1996a, 1996b), highlighting the effectiveness of the stratification based on geomorphology and soils to capture landscape heterogeneity (Zoneveld, 1989). Many liana studies conducted in Neotropical rainforests have measured stems at breast height (DBH, i.e., 1.3 m from the ground or roots) in sampling liana individuals (Alves et al., 2012; Burnham & Romero‐Saltos, 2015; Mascaro et al., 2004; Schnitzer et al., 2012). Although we used basal stem diameter, overall liana density (807.3 ha^−1^) and basal area (0.88 ha^−1^) at LTBS fell within the range reported by those studies considering lianas with DBH ≥1 cm (422–943 ind. ha^−1^ for density, and 0.5–1.9 m^2^ ha^−1^ for basal area). Some authors have reported exceptionally higher liana densities for other Neotropical rainforest as in Yasuní, Ecuador (1559 ind. ha^−1^, Romero‐Saltos, 2011), Viruá, Brazil (1055 ind. ha^−1^, Nogueira et al., 2011), and Chajul, Mexico (1043 ind. ha^−1^, Ibarra‐Manríquez & Martínez‐Ramos, 2002). The differences between the liana community of Los Tuxtlas and those from these sites can be explained considering the northernmost Neotropical location of the LTBS; however, some climate variables also could influence liana density, as there is strong evidence that localities with high annual rainfall (>3000 mm) that undergo a short dry season (less than 3 months), such as our study site, tend to have lower liana densities (DeWalt et al., 2010, 2015; Parolari et al., 2020).

The floristic composition of the liana community in the LTBS is generally consistent with Neotropical patterns, although with some noteworthy peculiarities. Gentry (1991) reported that Apocynaceae, Bignoniaceae, Celastraceae (including Hippocrateaceae), Malpighiaceae, and Sapindaceae are the most speciose families of climber plants in the Neotropics, a pattern that holds in our study site and in many other Neotropical liana surveys (Burnham & Romero‐Saltos, 2015; Ibarra‐Manríquez & Martínez‐Ramos, 2002; Macía, 2011; Schnitzer et al., 2012). However, in those studies, Fabaceae often ranked high (frequently among the first three places) in terms of liana species richness, but this family had a lower floristic importance in our study, in agreement with the reports of Bongers et al. (1988) and Ek‐Rodríguez et al. (2022) in the same reserve.

The floristic richness of all above‐mentioned families is associated with their high abundance values in the LTBS, but the same is not necessarily true for the most speciose genera (*Passiflora*, *Fridericia*, *Paullinia*, and *Smilax*). Regarding the most abundant species, *Combretum laxum* and *Forsteronia acouci* exhibit the same condition in other Neotropical forests, that is, they often appear among the most abundant taxa in many tropical forests (e.g., Ibarra‐Manríquez & Martínez‐Ramos, 2002, Lacandon Forest, Mexico; Romero‐Saltos, 2011, Yasuní, Ecuador; Schnitzer et al., 2012, Barro Colorado Island, Panama). This result, along with the fact that these species had similar distributions across all LUs (Figure 4), suggests a successful ecological performance of both species in different environments (Burnham & Romero‐Saltos, 2015; Ek‐Rodríguez et al., 2022).

### 4.2 Land units as contrasting habitat combinations for lianas

Based on the recognition of discrete LUs potentially offering contrasting habitat conditions for lianas, in this study we were able to describe liana community attributes in the LTBS more accurately than previously done. The only two studies analyzing the liana communities in a 1‐ha plot each (Bongers et al., 1988; Ek‐Rodríguez et al., 2022) reported very similar densities (368 and 396 ind. ha^−1^, respectively). Despite the considerable size of these plots, they are relatively homogeneous and close to each other (ca. 400 m), and thus they only represent a particular (north‐west facing) area of the reserve, despite being located in different LUs (PD and SDS, respectively, see Ek‐Rodríguez et al., 2022). Therefore, it is not surprising that we detected higher variation in liana community structural attributes as we covered a much higher surface and, more importantly, greater environmental heterogeneity.

Differences in liana density and basal area among LUs only emerged when they were field weighted (Figure 3). Scaling up ecological data to real scales is critical for understanding ecological processes in real ecosystems (Turner & Gardner, 2015; Zoneveld, 1989). By doing this we found evidence that the environmental mosaic, which is not homogeneous, promotes variation in liana community structure, driven mainly by soils and topography. Although our findings are in line with previous studies using similar approaches (Addo‐Fordjour et al., 2014; DeWalt et al., 2006; Ibarra‐Manríquez & Martínez‐Ramos, 2002; Macía, 2011; Rocha et al., 2022), none of those studies did adjust the variation associated with the proportion of each habitat and the environmental heterogeneity. This analysis shows that integrating the proportion of the LUs as an additional factor of heterogeneity allows the detection of differences in liana community structure. Interestingly, our results concur with studies using landscape stratification to evaluate differences in community attributes for other life forms, namely trees and palms (Durán et al., 2006; Fayolle et al., 2012; Miranda‐Gallegos et al., 2023; Navarrete‐Segueda et al., 2021) at the landscape scale, and for delineating major land use categories at regional scales (García‐Aguirre et al., 2010), highlighting the effectiveness of this approach.

Our results support the hypothesis that the LUs distinguished in the LTBS represent potentially different habitats for individual liana species performance, with several factors explaining their association. For one, dominant species make a differential contribution to density and basal area in the different LUs. Five of these species have higher densities in LUs characterized by steep slopes and higher elevations (i.e., SDS and SSH), whereas 4 of the 15 species with higher basal area make their major structural contribution in LF. In contrast, other species displayed similar values for both variables across all LUs (Figure 4). Together, these results suggest that the individual liana species have differential responses to the heterogeneous environmental conditions existing across this landscape, rather than showing a single response as a whole community to these conditions.

In addition, the NMDS showed a non‐random distribution of the species across LUs, which may also be explained by the heterogeneous environmental conditions that they capture. The first segregation factor in the NMDS ordinations based on species presence and density was slope steepness and elevation, as SDS, SSH, and SSL were clearly segregated from PD and LF on the ordination space (Figure 5a,b). These two latter LUs differ from each other in available water holding capacity and soil rooting depth (Table 1), which may in turn explain their segregation. Our results are consistent with previous studies on useful trees (Navarrete‐Segueda et al., 2021) and palms (Miranda‐Gallegos et al., 2023) in the same plots. For this latter study, the authors modeled climatic data describing a precipitation gradient related to changes in elevation even over small distances in the LTBS and showed the important role of these gradients in palm community differentiation, along with variation in soil water availability. Thus, these environmental variables are interrelated in a complex way and may be key drivers of plant community differentiation, as discussed in other liana studies (Liu et al., 2021, 2023; Manzané‐Pinzón et al., 2018; Rocha et al., 2022; Romero‐Saltos, 2011). Interestingly, the basal area data produced a contrasting ordination (Figure 5c), suggesting that environmental heterogeneity influences the establishment and biomass accumulation differentially among species.

Finally, SDS and LF, which contrast in slope steepness and water holding capacity (Table 1), had the lowest (8.9%) and the highest (32%) proportion of total liana individuals ≥10 cm in basal diameter, respectively. Ek‐Rodríguez et al. (2022) only reported two individuals with comparable diameters in a 1‐ha plot that was established in SDS. These two environmental factors are key for understanding liana community variation. Areas where soils have higher water holding capacity are more suitable for liana growth, but slope steepness may increase tree fall probability, ultimately prompting liana proliferation (Addo‐Fordjour et al., 2014; Liu et al., 2021; Schnitzer, 2018). These environmental factors also influence tree community structure and can jointly regulate liana establishment and stem growth (Manzané‐Pinzón et al., 2018; Medina‐Vega et al., 2022, 2021).

The liana community in the LTBS and the environmental factors that shape its attributes are very complex and, consequently, one should not expect to explain all the environmental heterogeneity solely on the basis of landscape stratification. Future studies should evaluate the effects of other factors, particularly the influence of continuous environmental variables (e.g., fine‐scale topographic features, soil physico‐chemical properties, tree community structure, light availability) on local composition and structure of lianas. Our study highlights the need to account for the heterogeneity in abiotic factors such as topography and soil features, as they inherently influence variation in plant performance (Florinsky, 2016; Hulshof & Spasojevic, 2020). Individual liana performance is thought to be mainly affected by stand structural attributes such as canopy gaps and tree density (Schnitzer, 2018). However, an increasing number of studies supports the idea that topo‐edaphic variation also influence liana community structure both directly, by affecting liana establishment and survival, and indirectly by affecting trellis and light availability (Addo‐Fordjour et al., 2014; Liu et al., 2023, 2021).

## 5 CONCLUSIONS

Our approach based on the distinction of discrete land units was useful to capture an important proportion of the environmental heterogeneity in the landscape of the LTBS. In this way, we were able to characterize more accurately the variation in liana community composition and structure, and their associated environmental heterogeneity. Differences in liana community attributes across the landscape are satisfactorily explained by topography and soils, with slope steepness, elevation, and soil water holding capacity emerging as key variables that together provide contrasting habitat combinations for lianas. Although other factors may also be related to liana community assembly (e.g., stand composition and structure), topography and soils may also co‐vary with them, influencing the distribution of lianas both directly and indirectly. In the future, an increasing ability to describe and measure the complexity of the environment in tropical forests and its spatial variation will allow a better understanding of the liana communities and their heterogeneity in these highly diverse ecosystems.

## AUTHOR CONTRIBUTIONS

**Iván Leonardo Ek‐Rodríguez:** Conceptualization (equal); data curation (equal); formal analysis (equal); investigation (equal); methodology (equal); writing – original draft (lead); writing – review and editing (equal). **Jorge A. Meave:** Conceptualization (equal); investigation (equal); writing – original draft (lead); writing – review and editing (equal). **Armando Navarrete‐Segueda:** Conceptualization (equal); investigation (equal); methodology (equal); writing – review and editing (equal). **M. Lourdes González‐Arqueros:** Investigation (equal); methodology (equal); writing – review and editing (equal). **Guillermo Ibarra‐Manríquez:** Conceptualization (equal); investigation (equal); methodology (equal); writing – review and editing (equal).

## CONFLICT OF INTEREST STATEMENT

The authors declare no conflicts of interest.

## Data Availability

The data that support the findings of this study are openly available in Dryad at https://doi.org/10.5061/dryad.9kd51c5rf.

## APPENDIX 1

**TABLE A1:** Checklist of liana species recorded in a survey conducted in the tropical rainforest at Los Tuxtlas Tropical Biology Station, Veracruz, Mexico.

Family/species	RD (%)	RBA (%)	*F* (No. of plots)
Acanthaceae (1/1)			
*Mendoncia retusa* Turrill	0.69	0.24	5
Amaranthaceae (1/1)			
*Chamissoa altissima* (Jacq.) Kunth	0.17	0.23	7
Apocynaceae (5/7)			
*Blepharodon mucronatum* (Schltdl.) Decne.	0.05	0.02	1
*Blepharodon* sp.	0.02	0.02	1
*Forsteronia acouci* (Aubl.) A.DC.	**5.90** ^**4**^	**7.92** ^**2**^	**15** ^**1**^
*Matelea magnifolia* (Pittier) Woodson	0.55	0.10	4
*Metalepis peraffinis* (Woodson) Morillo	0.05	0.00	1
*Prestonia mexicana* A.DC.	0.33	0.11	7
*P. portobellensis* (Beurl.) Woodson	0.02	0.00	1
Aristolochiaceae (1/2)			
*Aristolochia grandiflora* Sw.	0.02	0.00	1
*A. ovalifolia* Duch.	1.11	0.57	5
Asteraceae (4/5)			
*Mikania hookeriana* DC.	1.45	1.72	6
*M. leiostachya* Benth.	0.02	0.01	1
*Piptocarpha chontalensis* Baker	0.07	0.46	1
*Sinclairia discolor* Hook. & Arn.	0.02	0.02	1
*Tuxtla pittieri* (Greenm.) Villaseñor & Strother	0.40	1.22	6
Bignoniaceae (10/17)			
*Amphilophium crucigerum* (L.) L.G.Lohmann	0.05	0.05	2
*A. paniculatum* (L.) Kunth	0.18	1.52	2
*Anemopaegma chrysanthum* Dugand	1.12	0.48	9
*Bignonia binata* Thunb.	0.05	0.00	2
*B. hyacinthina* (Standl.) L.G.Lohmann	0.03	0.15	1
*Callichlamys latifolia* (Rich.) K.Schum.	0.89	1.54	12
*Cydista potosina* (K.Schum. & Loes.) Loes.	0.13	0.06	2
*Dolichandra uncata* (Andrews) L.G.Lohmann	0.03	0.26	1
*D. unguis‐cati* (L.) L.G.Lohmann	0.18	0.48	4
*Fridericia candicans* (Rich.) L.G.Lohmann	0.02	0.01	1
*F. chica* (Bonpl.) L.G.Lohmann	0.92	2.05	6
*F. florida* (DC.) L.G.Lohmann	0.99	1.63	7
*F. schumanniana* (Loes.) L.G.Lohmann	0.66	2.36	5
*Mansoa hymenaea* (DC.) A.H.Gentry	0.10	0.14	2
*M*. *verrucifera* (Schltdl.) A.H.Gentry	0.94	1.01	10
*Paragonia pyramidata* (Rich.) Bureau	0.73	1.13	10
*Stizophyllum riparium* (Kunth) Sandwith	1.26	0.56	6
Cannabaceae (1/1)			
*Celtis iguanaea* (Jacq.) Sarg.	0.58	1.57	10
Celastraceae (5/6)			
*Celastrus vulcanicolus* Donn.Sm.	1.70	1.26	8
*Hippocratea volubilis* L.	0.08	0.02	3
*Pristimera caribaea* (Urb.) A.C.Sm.	0.28	0.71	5
*P*. *celastroides* (Kunth) A.C.Sm.	**7.18** ^**3**^	**2.73** ^**10**^	**14** ^**2**^
*Salacia cordata* (Miers) Mennega	**11.79** ^**1**^	**10.34** ^**1**^	**15** ^**1**^
*Tontelea hondurensis* A.C.Sm.	0.40	0.23	8
Combretaceae (1/1)			
*Combretum laxum* Jacq.	**4.56** ^**5**^	**4.00** ^**5**^	**14** ^**2**^
Connaraceae (1/1)			
*Connarus schultesii* Standl.	**8.52** ^**2**^	**3.35** ^**8**^	**15** ^**1**^
Convolvulaceae (2/3)			
*Ipomoea indica* (Burm.) Merr.	0.08	0.07	4
*I*. *philomega* (Vell.) House	**2.69** ^**10**^	0.57	**14** ^**2**^
*Itzaea sericea* (Standl.) Standl. & Steyerm.	0.64	0.37	5
Cucurbitaceae (2/3)			
*Cionosicys macranthus* (Pittier) C.Jeffrey	0.07	0.01	3
*Cionosicyos* sp.	0.15	0.02	4
*Sicydium schiedeanum* Schltdl.	0.02	0.00	1
Dichapetalaceae (1/1)			
*Dichapetalum donnell‐smithii* Engl.	**2.81** ^**8**^	**4.26** ^**4**^	**14** ^**2**^
Dilleniaceae (2/2)			
*Doliocarpus dentatus* (Aubl.) Standl.	0.12	0.75	2
*Tetracera volubilis* L.	0.76	1.51	6
Dioscoreaceae (1/3)			
*Dioscorea composita* Hemsl.	0.02	0.00	1
*D*. *convolvulacea* Schltdl. & Cham.	0.15	0.02	5
*D*. *floribunda* M.Martens & Galeotti	0.02	0.00	1
Ehretiaceae (1/1)			
*Rochefortia lundellii* Camp	0.02	0.14	1
Euphorbiaceae (3/3)			
*Dalechampia magnistipulata* G.L.Webster & Armbr.	0.03	0.00	2
*Plukenetia volubilis* L.	0.02	0.01	1
*Tragia baroniana* Prain	0.05	0.00	2
Fabaceae (3/4)			
*Machaerium cobanense* Donn.Sm.	0.46	**3.59** ^**6**^	8
*M*. *floribundum* Benth.	1.11	**3.46** ^**7**^	9
*Oxyrhynchus trinervius* (Donn.Sm.) Rudd	0.18	0.03	4
*Senegalia hayesii* (Benth.) Britton & Rose	0.03	0.14	2
Heliotropiaceae (1/1)			
*Tournefortia hirsutissima* L.	0.07	0.03	2
Hernandiaceae (1/1)			
*Sparattanthelium amazonum* Mart.	0.28	0.94	4
Loganiaceae (1/2)			
*Strychnos panamensis* Seem.	0.79	0.21	11
*Strychnos* sp.	0.35	0.96	1
Malpighiaceae (6/7)			
*Heteropterys laurifolia* (L.) A.Juss.	0.21	0.35	3
*Hiraea fagifolia* (DC.) A.Juss.	1.64	0.75	8
*H*. *smilacina* Standl.	0.41	0.06	3
*Mascagnia vacciniifolia* Nied.	0.02	0.00	1
*Psychopterys rivularis* (C.V.Morton & Standl.) W.R.Anderson & S.Corso	2.58	1.61	10
*Stigmaphyllon lindenianum* A. Juss.	0.46	0.58	9
*Tetrapterys tinifolia* Triana & Planch.	0.78	0.71	10
Marcgraviaceae (3/3)			
*Marcgravia mexicana* Gilg	0.23	0.41	5
*Ruyschia enervia* Lundell	0.03	0.04	1
*Souroubea loczyi* (V.A.Richt.) de Roon	0.05	0.10	2
Menispermaceae (3/3)			
*Abuta panamensis* (Standl.) Krukoff & Barneby	1.57	**5.66** ^**3**^	**15** ^**1**^
*Disciphania calocarpa* Standl.	0.31	0.08	5
*Odontocarya mexicana* Barneby	1.07	0.27	8
Nyctaginaceae (1/1)			
*Pisonia aculeata* L.	1.06	2.02	10
Passifloraceae (1/6)			
*Passiflora ambigua* Hemsl.	0.07	0.05	2
*P*. *conzattiana* Killip	0.02	0.00	1
*P*. *hahnii* (E. Fourn.) Mast.	2.38	0.87	**14** ^**2**^
*P*. *helleri* Peyr.	0.03	0.02	2
*P*. *ligularis* Juss.	0.21	0.13	4
*P*. *microstipula* L.E.Gilbert & J.M.MacDougal	0.03	0.03	2
Petiveriaceae (1/1)			
*Trichostigma octandrum* (L.) H. Walter	0.41	0.77	7
Rhamnaceae (1/1)			
*Gouania lupuloides* (L.) Urb.	0.83	1.62	10
Rubiaceae (1/1)			
*Randia retroflexa* Lorence & M.Nee	1.55	0.70	**15** ^**1**^
Sapindaceae (3/7)			
*Paullinia clavigera* Schltdl.	**4.01** ^**7**^	1.46	**15** ^**1**^
*P*. *costaricensis* Radlk.	0.23	0.54	3
*P*. *costata Schltdl*. & Cham.	0.53	0.55	3
*P*. *venosa* Radlk.	0.38	0.46	9
*Serjania goniocarpa* Radlk.	0.07	0.01	2
*S*. *mexicana* (L.) Willd.	0.78	1.87	8
*Thinouia myriantha* Triana & Planch.	0.69	1.69	10
Schlegeliaceae (1/1)			
*Schlegelia nicaraguensis* Standl.	0.07	0.13	1
Smilacaceae (1/4)			
*Smilax aristolochiifolia* Mill.	0.23	0.06	7
*S*. *domingensis* Willd.	2.10	1.15	7
*S*. *regelii* Killip & C.V.Morton	0.28	0.07	2
*S*. *spinosa* Mill.	0.30	0.06	1
Solanaceae (4/5)			
*Juanulloa mexicana* (Schltdl.) Miers	0.17	0.18	3
*Lycianthes purpusii* (Brandegee) Bitter	0.55	0.36	12
*Solandra maxima* (Sessé & Moc.) P.S. Green	0.10	0.25	2
*Solanum dulcamaroides* Dunal	0.02	0.01	1
*S*. *wendlandii* Hook. f.	0.10	0.08	3
Urticaceae (1/1)			
*Urera lianoides* A.K.Monro & Al.Rodr.	0.73	0.21	6
Vitaceae (2/3)			
*Cissus gossypiifolia* Standl.	**4.41** ^**6**^	**2.91** ^**9**^	**14** ^**2**^
*C*. *microcarpa* Vahl	**2.77** ^**9**^	1.84	**13** ^**3**^
*Vitis tiliifolia* Humb. & Bonpl. ex Schult.	0.43	1.92	8

*Note*: Families are sorted alphabetically, and the number of genera and species in each of them are indicated in parenthesis. Species are also sorted alphabetically within their family, and for each of them, relative density (RD), relative basal area (RBA), and absolute frequency (*F*) are given. These values are indicated in bold typeface for the first 10 species having the highest density and basal area values, and with frequency ≥13 plots; for these cases, their ranks for each structural variable are indicated in superscripts.

**TABLE A2:** The 10 most important families according to their species richness and abundance.

Family	LF	PD	SDS	SSL	SSH	Total	Cumulative percentage
Species richness							
Bignoniaceae	10	11	12	8	8	17	15.5
Apocynaceae	3	3	2	5	2	7	21.8
Malpighiaceae	4	5	6	5	5	7	28.2
Sapindaceae	4	5	6	6	6	7	34.5
Celastraceae	5	5	6	5	4	6	40.0
Passifloraceae	3	4	4	3	2	6	45.5
Asteraceae	2	2	3	2	2	5	50.0
Solanaceae	2	2	2	4	1	5	54.5
Fabaceae	2	4	4	2	2	4	58.2
Smilacaceae	2	2	4	2	2	4	61.8
Abundance							
Celastraceae	105	162	403	198	430	1298	21.4
Connaraceae	128	80	90	93	125	516	30.0
Bignoniaceae	109	151	94	40	108	502	38.2
Vitaceae	30	60	119	128	124	461	45.9
Apocynaceae	104	66	93	98	57	418	52.8
Sapindaceae	39	84	88	114	80	405	59.5
Malpighiaceae	41	29	101	96	102	369	65.5
Combretaceae	21	24	88	61	82	276	70.1
Convolvulaceae	24	84	36	23	40	207	73.5
Menispermaceae	22	23	50	33	51	179	76.5

*Note*: Families are sorted in decreasing order according to their total values for each attribute. Species richness and abundance by family in each land unit (1.5 ha) and the total cumulative percentage (7.5 ha) are shown. Land units: LF, Lava flows; PD, Piedmonts; SDS, Steeply dissected slopes; SSH, Strongly undulating slopes of higher elevation; SSL, Strongly undulating slopes of lower elevation.

**TABLE A3:** The five most important genera according to their species richness and abundance.

Genera	LF	PD	SDS	SSL	SSH	Total	Cumulative percentage
Species richness							
*Passiflora*	3	4	4	3	2	6	5.5
*Fridericia*	2	3	4	3	1	4	9.1
*Paullinia*	2	3	3	3	4	4	12.7
*Smilax*	2	2	4	2	2	4	16.4
*Dioscorea*	2	0	1	1	1	3	19.1
Abundance							
*Salacia*	80	130	109	122	273	714	11.8
*Connarus*	128	80	90	93	125	516	20.3
*Pristimera*	23	21	274	71	63	452	27.8
*Cissus*	28	60	105	125	117	435	35.0
*Forsteronia*	86	45	92	80	54	357	40.9

*Note*: Genera are sorted in decreasing order according to their total values for each attribute. Species richness and abundance by genus in each land unit (1.5 ha) and the total cumulative percentage (7.5 ha) are shown. Land units: LF, Lava flows; PD, Piedmonts; SDS, Steeply dissected slopes; SSH, Strongly undulating slopes of higher elevation; SSL, Strongly undulating slopes of lower elevation.

**TABLE A4:** Summary of the general linear models for structural attributes.

Structural attribute (response variable)	Residual deviance (%)	*F*_10,14_	*p*‐value
Relative density	59.1	1.72	.22
Basal area (m^2^ ha^−1^)	66.3	1.27	.34
Mean diameter (cm)	62.0	1.53	.26
Maximum diameter (cm)	44.3	3.10	.06
Field weighted density	26.5	6.9	.006
Field weighted basal area	13.6	15.8	.0002

*Note*: The gaussian family were used in each model and the land unit was set as the explanatory variable. Function “ANOVA” were used to obtain the statistical parameters.
